# Mad Honey Intoxication: A Case Report

**DOI:** 10.7759/cureus.80631

**Published:** 2025-03-15

**Authors:** Matthew J Van Ligten, Ryan Gassner, Amogh Havanur, Wayne A Martini, Jessica Komara

**Affiliations:** 1 Emergency Medicine, Mayo Clinic Alix School of Medicine, Phoenix, USA; 2 Internal Medicine, Mayo Clinic, Phoenix, USA; 3 Emergency Medicine, Mayo Clinic, Phoenix, USA

**Keywords:** emergency medicine, grayanotoxins, mad honey, toxicology, treatment

## Abstract

A 49-year-old woman with a history of HIV and hypothyroidism developed symptomatic hypotension, bradycardia, and gastrointestinal distress after consuming honey she had purchased during a recent trip to Nepal. Her travel history and symptoms raised concerns about mad honey intoxication. Her condition improved with supportive care, including IV fluids, and atropine was not required for bradycardia. This case underscores the importance of considering mad honey toxicity in patients with unexplained cardiovascular symptoms and a history of honey ingestion. As interest in natural and traditional medicines grows, raising awareness about their potential risks becomes increasingly essential.

## Introduction

Mad honey intoxication is a rare toxicological phenomenon resulting from the ingestion of honey contaminated with grayanotoxins. These potent toxins, found in the nectar and pollen of certain *Rhododendron* species [1-3], are incorporated into honey when bees forage on these plants, producing what is commonly referred to as “mad honey” or “wild honey.” More than 20 different types of grayanotoxins have been identified in mad honey, and both the required dose and the specific symptoms vary seasonally [2]. However, further research is needed to clarify these variations, as most studies to date have been retrospective [2].

Although the dangers of grayanotoxins are not widely recognized, *Rhododendron* species and their honey are native to several mountainous and rural regions worldwide, including Nepal, Turkey, India, Brazil, parts of South America, Europe, Japan, and some areas of North America [1,4-6]. Many cultures have traditionally used mad honey in naturopathic medicine to treat conditions such as gastritis, ulcers, arthritis, hypertension, diabetes, infections, colds, and even sexual dysfunction [1,2,6,7]. Interestingly, some studies suggest that mad honey derived from *Rhododendron* species exhibits antiradical bioactivity, anti-inflammatory effects, and analgesic properties [2].

Despite these potential therapeutic effects and long-standing cultural practices, grayanotoxins pose a significant health risk. They primarily affect the cardiovascular and gastrointestinal systems by binding to voltage-gated sodium channels, leading to continuous cell depolarization and increased vagal nerve activation. This results in symptoms such as hypotension, bradycardia, syncope, and rhythm disturbances, including atrioventricular block [1,4]. Common gastrointestinal symptoms include dizziness, nausea, vomiting, and blurred vision [1,4,6]. While mad honey intoxication can cause severe symptoms, it is rarely fatal, and most cases resolve with supportive care within 24-48 hours [1,2]. Treatment typically involves IV fluids and atropine to manage hypotension and bradycardia, although severe cases may require transcutaneous pacing.

This case report describes a 49-year-old woman with a history of HIV and hypothyroidism who developed symptomatic hypotension, bradycardia, and nausea shortly after consuming honey acquired in Nepal. Her symptoms were managed conservatively with supportive care, without the need for atropine. This case highlights the clinical presentation of mad honey toxicity and underscores the importance of recognizing this condition in patients with unexplained cardiovascular symptoms following recent honey ingestion, particularly among travelers to endemic regions.

## Case presentation

A 49-year-old woman with a history of HIV (undetectable viral load on a combined regimen of bictegravir 50 mg, emtricitabine 200 mg, and tenofovir 25 mg daily) and hypothyroidism presented to the ED after returning from a trip to Nepal. She reported lightheadedness, head pressure, nausea, and flushing. Her symptoms began after she ingested two cups of tea containing honey purchased during her trip. Initially, she felt well enough to go to the gym; however, upon arrival, her symptoms worsened, prompting her to seek medical attention.

**Figure 1:**
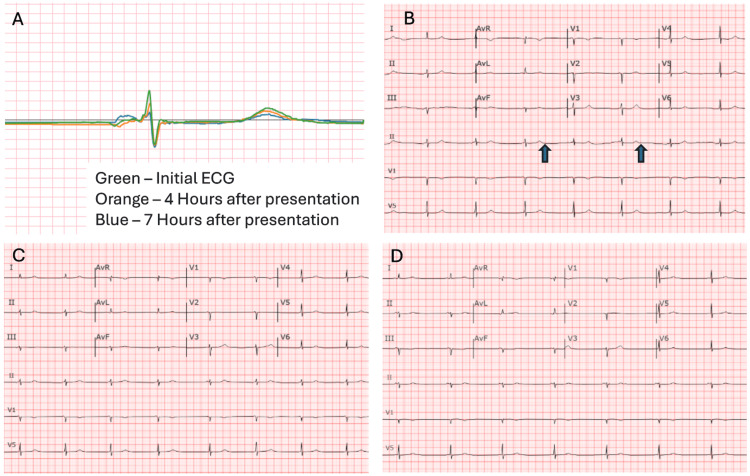
Overlay of the three ECG tracings taken at different time points (A) - green represents the initial ECG, orange corresponds to four hours after presentation, and blue represents seven hours post-presentation — demonstrating progressive changes in the QRS complex and ST segments. The serial ECGs demonstrate a progression of conduction changes over time, starting with an ectopic atrial bradycardia (B), asynchronous p waves present (blue arrows) evolving into junctional bradycardia (C) four hours after initial presentation, and eventually transitioning to sinus rhythm (D) seven hours after presentation. Initially, the ectopic atrial rhythm is characterized by an unusual P-wave axis and low heart rate, suggesting an atrial focus outside the SA node. By the second ECG, the rhythm shifts to junctional bradycardia, evidenced by a short PR interval and absent or retrograde P waves, indicating pacemaker activity from the AV junction. Additionally, LAD, low-voltage QRS, and an anteroseptal infarct pattern become evident, suggesting underlying structural or ischemic heart disease. The last ECG shows a notable improvement that occurs with the restoration of sinus rhythm, resolution of ST depression in the anterior leads, and shortening of the QT interval, indicating improved AV nodal conduction and repolarization dynamics. These changes suggest a transient conduction disturbance. AV, atrioventricular; LAD, left axis deviation; SA, sinoatrial

In the ED, her vital signs were as follows: blood pressure: 82/44 mmHg; temperature: 36.6°C; heart rate: 47 beats per minute; respiratory rate: 20 breaths per minute; and oxygen saturation: 100% on room air. A CBC showed an elevated hematocrit (48%) and hemoglobin (16.4 g/dL) but no leukocytosis. A CMP revealed mildly elevated creatinine (1.10 mg/dL) and calcium (10.4 mg/dL). The initial ECG showed sinus bradycardia with possible ectopic atrial bradycardia and a QTc of 467 ms (Figure 1).

Toxicology was consulted, and given her recent travel history, they suspected mad honey intoxication due to grayanotoxins. Upon further discussion, the patient also reported experiencing diarrhea, nausea, and vomiting - symptoms consistent with the cholinergic-like effects of mad honey, despite the absence of classic cholinergic signs. Notably, she confirmed that she had consumed the honey for the first time that day, using it as a “supplement” in her tea. She had purchased it from a medicinal store in Nepal.

Based on this information, she received 2 L of normal saline, which led to a transient improvement in blood pressure. Continuous telemetry was initiated, and atropine was reserved for symptomatic bradycardia if needed. The consulting internist evaluated the patient and recommended admission to the progressive care unit, a higher level of care that provides increased monitoring and telemetry compared to standard admission floors.

During her two-day hospital stay, her blood pressure gradually improved with supportive care, and her symptoms resolved. Serial ECGs showed a progression from sinus bradycardia to a junctional rhythm with left axis deviation before eventually returning to sinus rhythm with a shortened QT interval compared to her initial presentation (Figure 1). She remained under continuous telemetry monitoring but did not experience any episodes of symptomatic bradycardia. By hospital day 2, her blood pressure had stabilized, and she was discharged in stable condition. She was advised to avoid further ingestion of the honey and to return to the ED if her symptoms recur.

## Discussion

Mad honey intoxication is a distinct toxicological syndrome that primarily affects the cardiovascular and gastrointestinal systems due to the presence of grayanotoxins in the honey [6]. These toxins bind to and persistently activate sodium channels, leading to symptoms such as dizziness, nausea, vomiting, blurred vision, hypotension, and bradycardia [3,6]. In more severe cases, grayanotoxin exposure can cause cardiac abnormalities, including atrioventricular block, sinus bradycardia, and atrial fibrillation. Neurological manifestations such as syncope or seizures may also occur due to central nervous system involvement [3,6].

**Figure 2:**
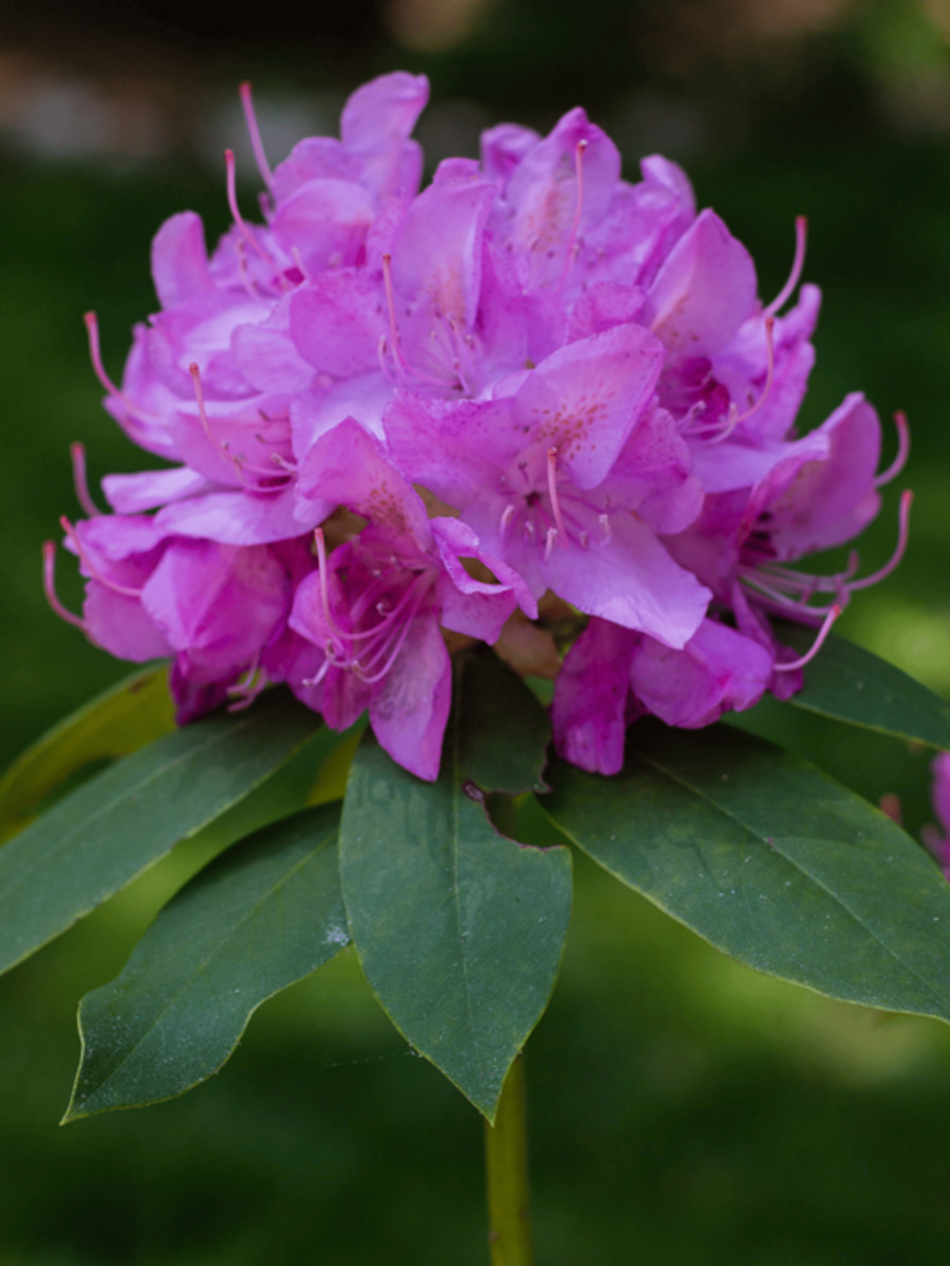
Rhododendron flowers that create the grayanotoxins that may contaminate the honey made in endemic areas Image source: Grayanotoxins: of rhododendrons and mad honey. (2014). Accessed: March 10, 2025: https://naturespoisons.com/2014/05/08/grayanotoxins-of-rhododendrons-and-mad-honey/ [8]

The severity of symptoms often correlates with the amount of honey consumed and the concentration of grayanotoxins, which can vary significantly. Several factors influence this variability, including the specific *Rhododendron* species (Figure 2) [8] from which the bees collected nectar, the honey’s geographical origin, and the season in which it was harvested. These fluctuations make it challenging to establish a clear dose-response relationship, as grayanotoxin concentrations in mad honey are inconsistent.

The diagnosis of mad honey intoxication is primarily based on clinical presentation and a history of honey consumption. Since no laboratory tests are readily available to quantify grayanotoxin levels, healthcare providers must rely on characteristic symptoms alongside a history of ingestion, particularly in travelers returning from endemic regions such as Nepal and Turkey [6].

Treatment is largely supportive. IV fluids help manage hypotension, while atropine, an anticholinergic agent, may be administered for persistent bradycardia. In severe cases, additional interventions such as vasopressors to maintain blood pressure and temporary cardiac pacing for rhythm abnormalities may be necessary [6]. However, in this case, the patient improved with conservative fluid resuscitation alone, without the need for atropine. Previous studies have shown that most cases of mad honey intoxication resolve within 24-48 hours, with bradycardia typically responding well to supportive care, making this case a representative example of the condition’s typical course and management [6].

The rising popularity of natural and traditional medicines has led to greater interest in products like mad honey, which has long been valued in certain cultures for its perceived medicinal properties. As more individuals turn to alternative remedies in an effort to avoid the side effects of conventional pharmaceuticals, natural products like mad honey are gaining traction among those seeking holistic approaches. However, these treatments are not without risks. This growing trend highlights the need for greater awareness of the potential dangers associated with such remedies. This case underscores the importance of considering mad honey intoxication in the differential diagnosis of unexplained hypotension and bradycardia, particularly in patients with recent travel to regions where it is commonly consumed for its purported health benefits.

## Conclusions

Mad honey intoxication, although rare, should be considered when patients present with unexplained hypotension and bradycardia, particularly if they have recently consumed honey from endemic regions. This case report highlights the clinical course and management of mad honey toxicity, which primarily involves supportive care and close monitoring. While symptoms can vary, classic findings include hypotension, dizziness, gastrointestinal distress, and bradycardia. Therefore, mad honey intoxication should be suspected in patients with the acute onset of these symptoms, especially when more common conditions - such as cardiac ischemia, deep vein thrombosis, infection, or hypoxia - are not evident.

As natural and traditional medicines continue to gain popularity, awareness of mad honey’s toxic potential is essential for both healthcare providers and the general public. Educating individuals about the risks associated with grayanotoxins, along with careful attention to a patient’s travel history, can facilitate early recognition and appropriate management, potentially preventing severe complications.
